# Regulation of Adipogenesis and Lipid Deposits by Collapsin Response Mediator Protein 2

**DOI:** 10.3390/ijms21062172

**Published:** 2020-03-21

**Authors:** Yih-Hsin Chang, Jen-Ning Tsai, Shu-Wen Chang, Wei-Ting Hsu, Ching-Ping Yang, Chiao-Wan Hsiao, Ming-Yuh Shiau

**Affiliations:** 1 Department of Biotechnology and Laboratory Science in Medicine, National Yang-Ming University, Taipei 112, Taiwan; cyh@ym.edu.tw (Y.-H.C.); g128525702@gmail.com (S.-W.C.); wei_ting0523@hotmail.com (W.-T.H.); d49407012@ym.edu.tw (C.-P.Y.); hcw_alicia_1990@hotmail.com (C.-W.H.); 2 Department of Medical Laboratory and Biotechnology, Chung Shan Medical University, Taichung 402, Taiwan; jeningts@csmu.edu.tw; 3 Clinical Laboratory, Chung Shan Medical University Hospital, Taichung 402, Taiwan; 4 Program in Molecular Medicine, National Yang-Ming University and Academia Sinica, Taipei 112, Taiwan; 5 Department of Nursing, College of Nursing, Hungkuang University, Taichung 433, Taiwan

**Keywords:** adipogenesis, collapsin response mediator protein 2, glycogen synthase kinase-3β, obesity, lipid metabolism, neurodegenerative disease

## Abstract

As emerging evidence suggesting neurodegenerative diseases and metabolic diseases have common pathogenesis, we hypothesized that the neurite outgrowth-controlling collapsin response mediator protein 2 (CRMP2) was involved in energy homeostasis. Therefore, putative roles of CRMP2 in adipocyte differentiation (adipogenesis) and lipid metabolism were explored and addressed in this study. CRMP2 expression profiles were in vitro and in vivo characterized during adipogenic process of 3T3-L1 pre-adipocytes and diet-induced obese (DIO) mice, respectively. Effects of CRMP2 on lipid metabolism and deposits were also analyzed. Our data revealed that CRMP2 expression pattern was coupled with adipogenic stages. CRMP2 overexpression inhibited cell proliferation at MCE phase, and significantly reduced lipid contents by down-regulating adipogenesis-driving transcription factors and lipid-synthesizing enzymes. Interestingly, GLUT4 translocation and the lipid droplets fusion were disturbed in CRMP2-silencing cells by affecting actin polymerization. Moreover, adipose CRMP2 was significantly increased in DIO mice, indicating CRMP2 is associated with obesity. Accordingly, CRMP2 exerts multiple functions in adipogenesis and lipid deposits through mediating cell proliferation, glucose/lipid metabolism and cytoskeleton dynamics. The present study identifies novel roles of CRMP2 in mediating adipogenesis and possible implication in metabolic disorders, as well as provides molecular evidence supporting the link of pathogenesis between neurodegenerative diseases and metabolic abnormalities.

## 1. Introduction

Global type 2 diabetes mellitus (T2DM) prevalence has been exponentially increasing over the past 3 decades. The rising T2DM epidemic represents one of the most serious threats to human health due to the premature morbidity and mortality caused by multiple diabetic complications. Insulin resistance is an early defect associated with obesity and T2DM. Nevertheless, the exact etiology leading to decreased insulin action is not completely resolved.

Insulin exerts its anabolic function through PI3K-Akt signaling pathway in the target cells. The insulin-derived Akt signaling triggers the inactivation of glycogen synthase kinase-3β (GSK-3β) which then promotes glycogen synthesis [1,2]. Elevated GSK-3β activity and expression are identified in obese and diabetic humans and rodents [3,4]. Inhibiting GSK-3β by small molecules or peptide inhibitors improves insulin action and glucose homeostasis [5]. In addition to serving as one of the major target molecules of insulin in modulating energy homeostasis, GSK-3β is the critical enzyme controlling microtubule dynamics via mediating the activity of collapsin response mediator protein 2 (CRMP2) [6,7,8].

CRMP2 is the most studied member among the CRMP family proteins, which regulates multiple physiological activities including microtubule dynamics, neuronal outgrowth and polarity, and vehicle transportation [6,7,8]. Extracellular signals, such as the inhibitory protein for axonal guidance Sema3A, activate CRMP2 through PI3K-Akt signaling pathway [9]. CRMP2 activity is mainly regulated by cyclin-dependent kinase 5 (Cdk5)- and GSK-3β-mediated phosphorylation [10], with phosphorylation at its C-terminal Ser522 by Cdk5 as the pre-requisite for the subsequent phosphorylation by GSK-3β at Ser518/Thr514/Thr509. The phosphorylation status of CRMP2 Thr514 is the predominant site determining microtubule-binding activity. 

CRMP2 is closely associated with neurodegenerative diseases such as Alzheimer’s disease (AD) and Parkinson’s disease (PD) [11]. CRMP2 hyperphosphorylation is characterized as an early event in AD progression, leading to the increase of amyloid precursor protein [12]. Suppressing hippocampal CRMP2 hyperphosphorylation in AD model ameliorates the cognitive dysfunction and hippocampal axon degeneration induced by beta-amyloid precipitation [13].

Accumulating evidence suggests a potential network composed of CRMP2, cytoskeleton, and GSK-3β is implicated in energy metabolism. First of all, CRMP2 regulates cell proliferation in mitotically active non-neuronal cells [7,10,14]. Secondly, CRMP2 is suggested to participate in T2DM pathogenesis based on its highly expression in pancreatic islet, as well as the clinical relationship and pathological similarity between AD and T2DM [15]. Thirdly, GSK-3β the major regulator controlling CRMP2 activities and thus microtubule dynamics in neuronal cells, is also the important insulin downstream signaling molecule modulating metabolic homeostasis. In addition, a recent finding from Nagano et al. revealed another member of the CRMP family protein, CRMP3/DPYSL4, plays a key role in the tumor-suppressor function of p53 by regulating oxidative phosphorylation and cellular energy supply [16]. The authors thus suggested that CRMP3/DPYSL4 is linked to the pathophysiology of both cancer and obesity, supporting our hypothesis regarding the involvement of CRMP in metabolic homeostasis.

We previously provide the molecular evidence addressing the linkage between PD and T2DM comorbidity by uncovering the roles of PTEN-induced kinase 1 (PINK1)-presenilin associated rhomboid-like protein (PARL)-PINK1-Parkin system in adipocyte differentiation and energy metabolism [17]. By combining the above clues, it is reasonable to hypothesize that GSK-3β serves as the convergence between insulin signaling and CRMP2 to regulate energy metabolism. However, putative role of CRMP2 in metabolic homeostasis has never been documented. In this context, the involvement of CRMP2 in glucose/lipid metabolism of 3T3-L1 adipocyte differentiation was investigated in this study. Our results demonstrate that CRMP2 exerts multiple functions in adipogenesis and determines lipid deposits through mediating cell proliferation, glucose/lipid metabolism, and cytoskeleton dynamics.

## 2. Results

### 2.1. Alteration of CRMP2 Expression Pattern during Adipogenesis

**Figure 1:**
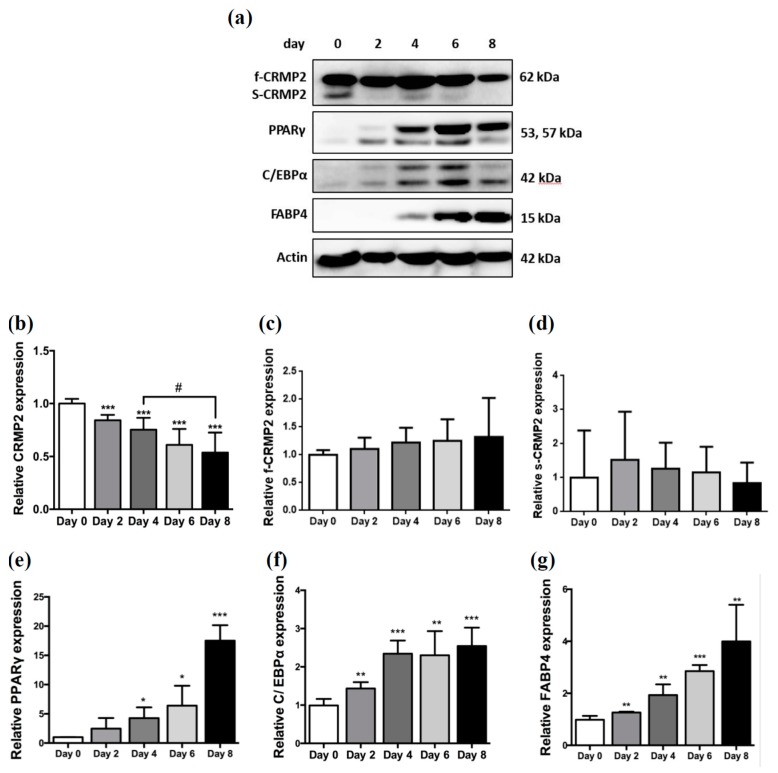
Alteration of collapsin response mediator protein 2 (CRMP2) expression pattern during adipogenesis. 3T3-L1 preadipocytes were induced into adipocytes by MDI cocktails. (**a**) CRMP2 was analyzed by Western blotting during adipogenesis. C/EBPα, PPARγ, and FABP4 were served as markers of adipocytes differentiation. (**b**–**g**) Relative expression of the detected proteins was quantified and normalized to actin. The data were presented as the mean ± SEM, and statistically analyzed by one-tailed unpaired Student *t*-test (*n* = 3). * *p* < 0.05 vs. day 0; ** *p* < 0.01 vs. day 0; *** *p* < 0.005 vs. day 0; # *p* < 0.05 vs. day 4.

CRMP2 protein expression was temporally examined during the entire process of 3T3-L1 adipocyte differentiation. Important proteins driving adipogenesis, including C/EBPα, PPARγ, and FABP4, were analyzed to ensure successful differentiation. While the full-length CRMP2 (f-CRMP2, ~62 kDa) remained rather consistent during the entire differentiation period, the expression of smaller CRMP2 (s-CRMP2, ~58 kDa) was decreasing along with the progression of adipogenesis to a barely detected level in mature adipocytes on day 8 (Figure 1a–d). 

CRMP2 mRNA remained consistent levels during adipogenesis. CRMP2 loses its tubulin-binding activity either when it is phosphorylated [18] or processed to generate s-CRMP2 by calpain-mediated proteolysis [19]. For examining if phosphorylation and/or proteolytic processing contributed to the alteration of CRMP2 expression profile during adipogenesis, the possible changes of CRMP2 expression in the presence of either lambda protein phosphatase (λ−PP) or calpain inhibitor ALLN (Ac-Leu-Leu-Nle-CHO) were analyzed. Interestingly, the addition of phosphatase or calpain inhibitor did not alter the CRMP2 expression pattern.

In neural cells, GSK-3β inactivates the tubulin-binding activity of CRMP2 by phosphorylating CRMP2 at Thr514 (pCRMP2 Thr514). The inactivated CRMP2 is then degraded [20], leading to the inhibition of axonogenesis and the collapse of neural growth cone [21,22]. While examining whether the decrease of s-CRMP2 during adipogenesis was resulted from protein degradation after being phosphorylated, pCRMP2 Thr514 was undetected once the cells were allowed to differentiate. The results support the previous finding that pCRMP2 is de-phosphorylated in response to contact inhibition-induced quiescence [14]. and imply that CRMP2 activity is required for adipocyte differentiation.

### 2.2. CRMP2 Overexpression Inhibits Adipogenesis

**Figure 2:**
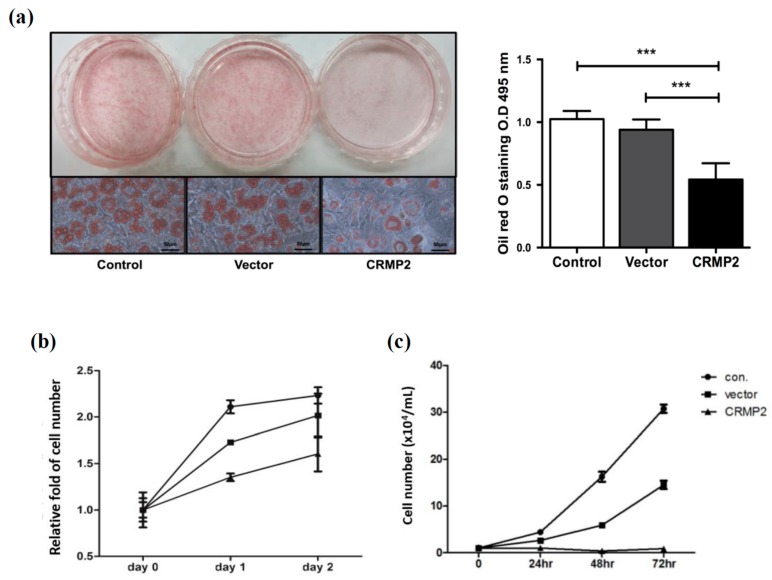
CRMP2 overexpression inhibits adipocyte differentiation and cell proliferation at mitotic clonal expansion (MCE) phase. Pre-adipocytes were transfected with either CRMP2-expressing or control vector on day-2, then allowed to enter differentiation. (**a**) Lipid contents in the mature adipocytes with CRMP2 overexpression (CRMP2-cells), control vector (Vector) and the mock transfection (Control) were measured by Oil-Red O staining on day 8. The images were taken using the Nikon ECLIPSE TS100 microscope with 40× objective, scale bars = 50 μm. (**b**) Cells in MCE phase were trypsinized and counted by trypan blue exclusion on the day indicated. (**c**) Pre-adipocytes were transfected with either CRMP2-expressing or control constructs, then trypsinized and counted on the indicated time without MDI induction. The data were presented as the mean ± SEM, and statistically analyzed by one-tailed unpaired Student *t-*test (*n* = 3). *** *p* < 0.005.

It is intriguing that while s-CRMP2 is gradually decreased, CRMP2 remains active during the adipogenic process. To address the effects of CRMP2 on adipogenesis, pre-adipocytes were transfected with f-CRMP2-expressing vector on day -2. Differentiation efficiency of cells with CRMP2 overexpression (CRMP2-cells), control vector (Vector) and the mock transfection (Control) was respectively evaluated by Oil-Red O staining. Interestingly, the lipid contents in CRMP2-cells were significantly decreased about 40% on day 8 (Figure 2a). 

When pre-adipocytes are induced to differentiate, they first undergo proliferation during the initial 48 hr (mitotic clonal expansion (MCE) phase), followed by differentiation phase to become mature adipocytes [23,24]. For dissecting the effect of CRMP2 on adipogenesis, cell proliferation of the CRMP2 transfectants at MCE phase was investigated. The results showed that numbers of CRMP2-cells at MCE phase were decreased about 30% (Figure 2b). Meanwhile, effect of CRMP2 overexpression on pre-adipocyte proliferation was also investigated. Cell proliferation was almost abolished by CRMP2 overexpression (Figure 2c). 

**Figure 3:**
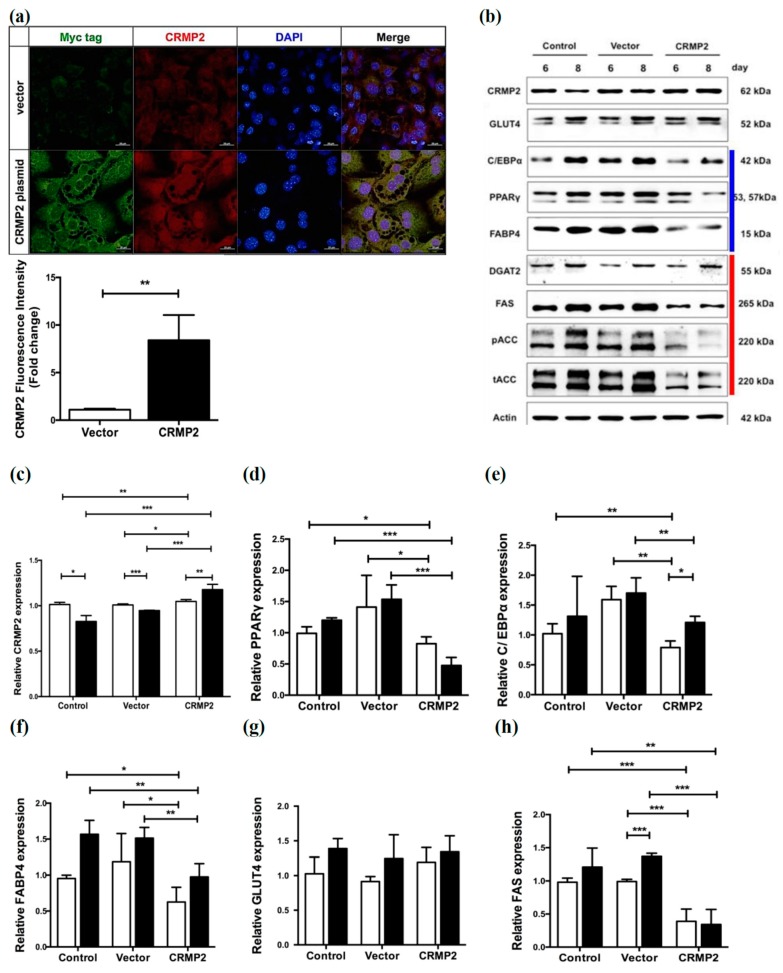

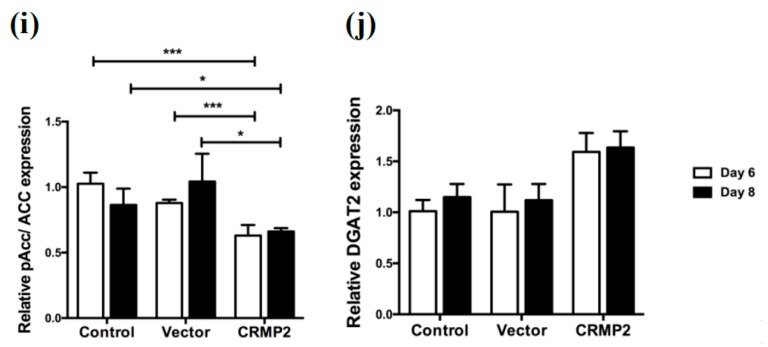
CRMP2 overexpression down-regulates the expression of critical adipogenic transcription factors and lipid-synthesizing enzymes. CRMP2-expressing or control vector were transfected into the cells on day 4 after entering differentiation. (**a**) Expression of CRMP2 was detected and quantified by immunofluorescence, scale bars = 20 μm. (**b**–**j**) Levels of CRMP2, adipogenic marker proteins including PPARγ, C/EBPα, GLUT4, and FABP4 and critical lipid-synthesizing enzymes on day 6 and 8 were analyzed by Western blotting, with actin as an internal control. Blue and red line at the right of panel (**b**) indicated proteins related to adipogenesis and lipid synthesis, respectively. The data were presented as the mean ± SEM, and statistically analyzed by one-tailed unpaired Student *t-*test (*n* = 3). * *p* < 0.05; ** *p* < 0.01; *** *p* < 0.005.

The effects of CRMP2 overexpression on late stage of adipogenesis after MCE phase were subsequently analyzed. CRMP2-expressing or control vector were transfected into the cells on day 4 after differentiation, then the important adipogenic proteins were analyzed on day 6 and 8. As shown in both immunofluorescent imaging and Western blotting (Figure 3a–c), CRMP2 levels were successfully elevated in CRMP2-transfectants. While PPARγ (Figure 3d), C/EBPα (Figure 3e) and FABP4 (Figure 3f) were significantly decreased, GLUT4 was not altered in CRMP2-cells (Figure 3g). Meanwhile, critical lipid-synthesizing enzymes for lipid accumulation, including FAS (Figure 3h), pACC and ACC (Figure 3i), were significantly reduced in CRMP2-cells. Downregulation of these important lipid-synthesizing enzymes is consistent with and, at least in part, explains the significantly reduced lipid contents in CRMP2-cells (Figure 2a). As the CRMP2 vector was transfected on day 4, the lipid synthesis-inhibitory activity of CRMP2 is independent to its cell proliferation-suppressing function at MCE stage.

**Figure 4:**
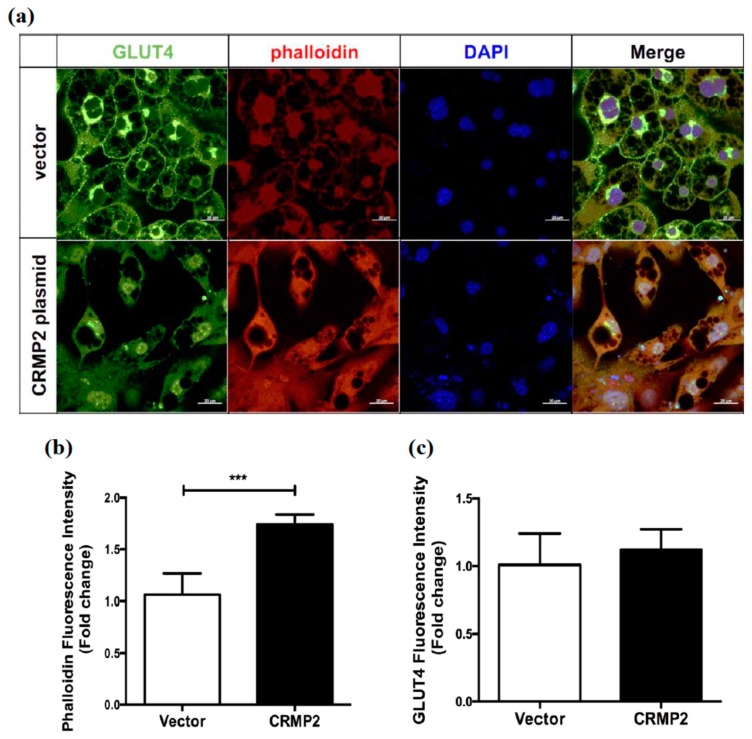
Effects of CRMP2 overexpression on cytoskeleton and GLUT4 expression. CRMP2-expressing or control vector were transfected into the cells on day 4 after entering differentiation. (**a**) Expression patterns of GLUT4 and phalloidin-positive actin filaments were detected and quantified in mature transfects on day 8 by immunofluorescence. The images were captured using a Zeiss LSM 880 laser confocal microscope, scale bars = 20 μm. The images RGB plots were measured and generated by ImageJ RGB profile plots. (**b**,**c**) Data were presented as means ± SEM of about 50 cells in each group, and analyzed by one-tailed unpaired Student *t*-test. *** *p* < 0.005.

We assumed that CRMP2 overexpression may cause the alteration of microtubule stability given CRMP2 regulates axonogenesis through modulating cytoskeleton dynamics [25], the GLUT4 translocation and fusion at cortical area in particular, which mediates energy metabolism via regulating glucose uptake ability. Therefore, the formation of phalloidin-positive polymerized F-actin was examined, with the above observations being further verified by microscopic strategy. Interestingly, CRMP2-cells exhibited more fibroblast-like morphology on day 8, rather than the signature rounded shape of mature adipocytes as their control counterparts (Figure 4a). Phalloidin-staining intensity was augmented about 2 folds in CRMP2-cells (Figure 4b), indicating actin polymerization in the cortical area is promoted. Consistent with the Western blotting data, the GLUT4 signal remained unchanged in CRMP2-cells (Figure 4c). 

The above results strongly suggest that CRMP2 suppresses adipocyte differentiation through inhibiting the machinery required for adipogenesis, including cell proliferation at MCE phase, the expression of critical adipogenic transcription factors leading to the decreased adipogenic markers, and the critical lipid-synthesizing enzymes resulting in the reduced lipid contents in the mature adipocytes. In addition, CRMP2 mediates actin dynamics which is crucial for the dramatic morphological remodeling of the fibroblastic pre-adipocytes transforming to the characterized round shapes of mature adipocytes.

### 2.3. CRMP2 Knockdown Reverses the Inhibitory Effects on Adipogenesis 

**Figure 5:**
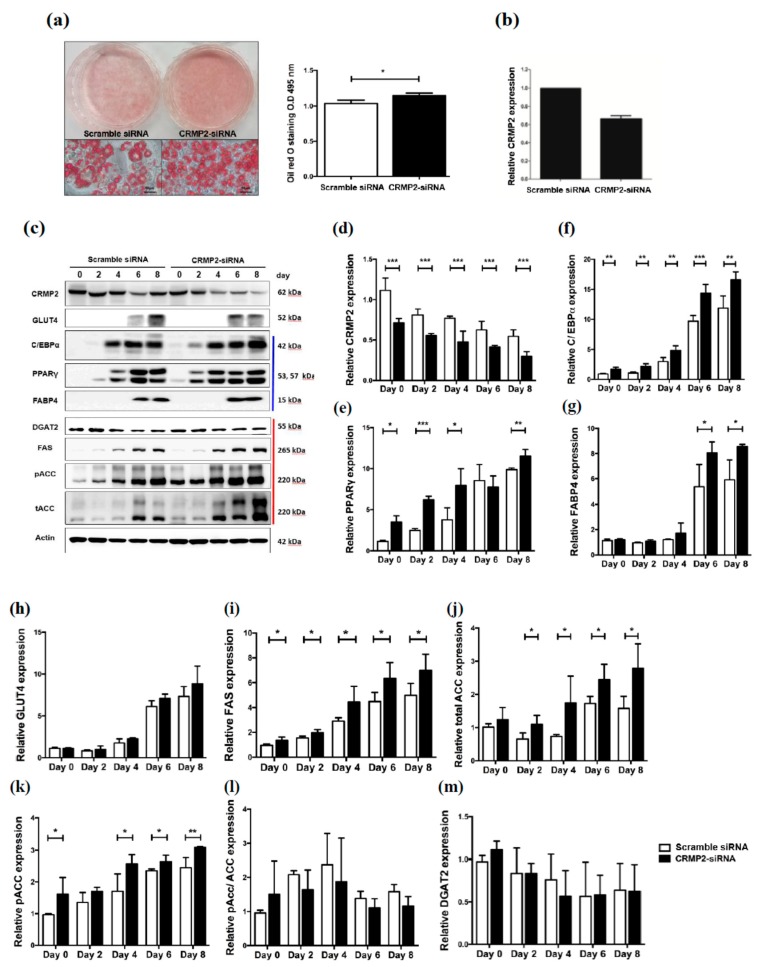
CRMP2 knockdown reverses the inhibitory effects on adipogenesis. Cells were transfected with CRMP2-specific siRNA (CRMP2-siRNA cells) or scramble siRNA on day-2, then subjected to differentiation. (**a**) Lipid accumulation in the mature adipocytes was measured and quantified as described, scale bars = 50 μm. Expression of CRMP2 RNA (**b**) and protein levels of CRMP2, PPARγ, C/EBPα, GLUT4 and FABP4 and critical lipid-synthesizing enzymes (**c**–**m**) were analyzed as the day indicated, with actin as an internal control. Blue and red line at the right of panel (**b**) indicated proteins related to adipogenesis and lipid synthesis, respectively. The data were presented as the mean ± SEM, and statistically analyzed by one-tailed unpaired Student *t-*test (*n* = 3). * *p* < 0.05; ** *p* < 0.01; *** *p* < 0.005.

The above findings demonstrate that CRMP2 inhibits adipogenesis through mediating cell growth, critical adipogenic-transcription factors, lipid synthesis and cytoskeleton dynamics. We presumed that these inhibitory effects would be reversed by CRMP2-specific siRNA, therefore, effects of CRMP2-silencing on adipogenesis were next analyzed. Cells were transfected with CRMP2-specific siRNA (CRMP2-siRNA cells) or scramble siRNA on day-2, then subjected to differentiation. As anticipated, lipid contents were increased in CRMP2-siRNA cells (Figure 5a) with lower CRMP2 levels (Figure 5b–d). PPARγ (Figure 5e), C/EBPα (Figure 5f) and FABP4 (Figure 5g) were significantly promoted while GLUT4 (Figure 5h) was still not affected. Moreover, FAS (Figure 5i), ACC (Figure 5j), and pACC (Figure 5k) were significantly up-regulated in CRMP2-siRNA cells, which paralleled to the corresponding increased lipid contents in mature CRMP2-siRNA adipocytes. 

**Figure 6:**
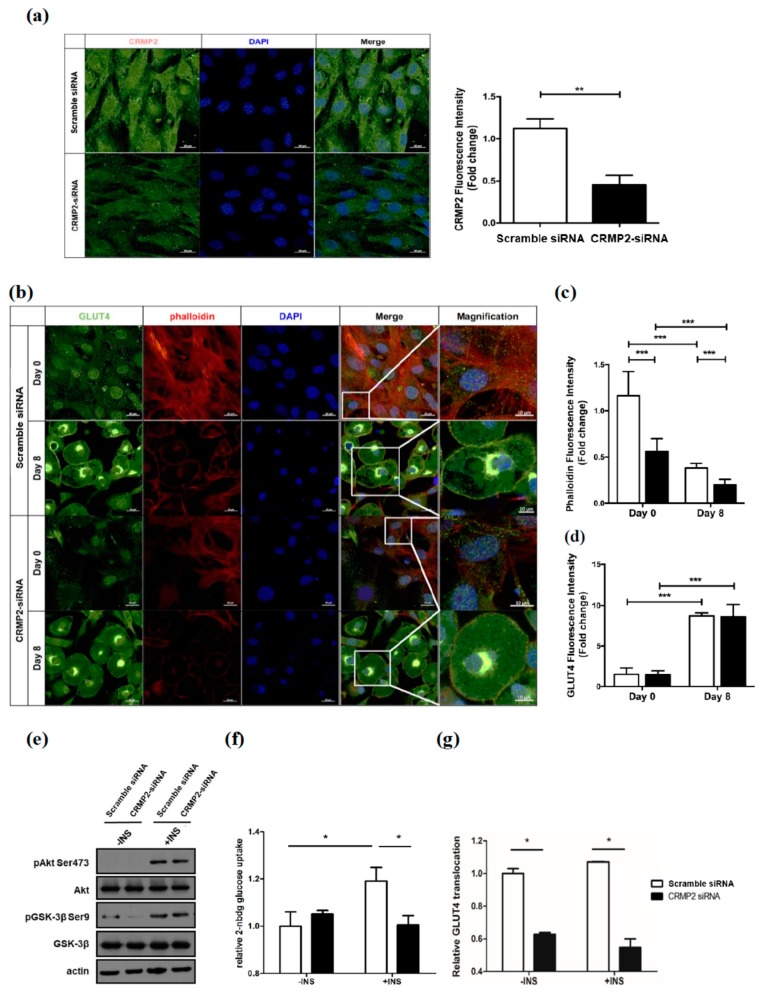
Effects of CRMP2 knockdown on cytoskeleton and glucose uptake. Cells were transfected with CRMP2-specific siRNA (CRMP2-siRNA cells) or control siRNA (scramble siRNA) on day -2, then subjected to differentiation. CRMP2 (**a**), GLUT4 and phalloidin-positive actin filaments (**b**–**d**) were detected and quantified in transfects on day 0/8 as described. The scale bars represented 20 μm in regular images and 10 μm in Magnification images, respectively. Data were presented as means ± SEM of about 50 cells in each group and analyzed by one-tailed unpaired Student *t*-test. Insulin-stimulated signaling molecules pAkt Ser 473 and pGSK-3β Ser 9 (**e**), 2-NBDG uptake (**f**), and GLUT4 translocation (**g**) were measured as described. The data were presented as the mean ± SEM, and statistically analyzed by two-tailed unpaired Student *t*-test (*n* = 3). * *p* < 0.05, ** *p* < 0.01, *** *p* < 0.005.

Microscopically, CRMP2 was decreased about 50% in CRMP2-siRNA transfectants (Figure 6a). Notably, the actin filaments were also significantly reduced about half in CRMP2-siRNA pre-adipocytes (day 0) and the mature adipocytes (day 8; Figure 6b,c). While GLUT4 expression (Figure 6d) and insulin-induced pAkt and pGSK-3β (Figure 6e) were not affected, insulin-stimulated glucose uptake was significantly decreased in CRMP2-siRNA cells (Figure 6f). It indicates that glucose uptake is down-regulated although insulin signaling and GLUT4 levels are intact in CRMP2-siRNA cells. We next tested if the suppressed insulin-stimulated glucose uptake was resulted from the inhibition of GLUT4 translocation. The data showed that the membrane GLUT4 ratio under insulin treatment was greatly decreased about 40% in CRMP2-siRNA cells (Figure 6g), indicating the decreased glucose uptake in CRMP2-siRNA cells is impaired due to, at least in part, the disturbed GLUT4 translocation rather than the intracellular GLUT4 levels.

### 2.4. CRMP2 Regulates Lipid Accumulation through Mediating Cytoskeleton

**Figure 7:**
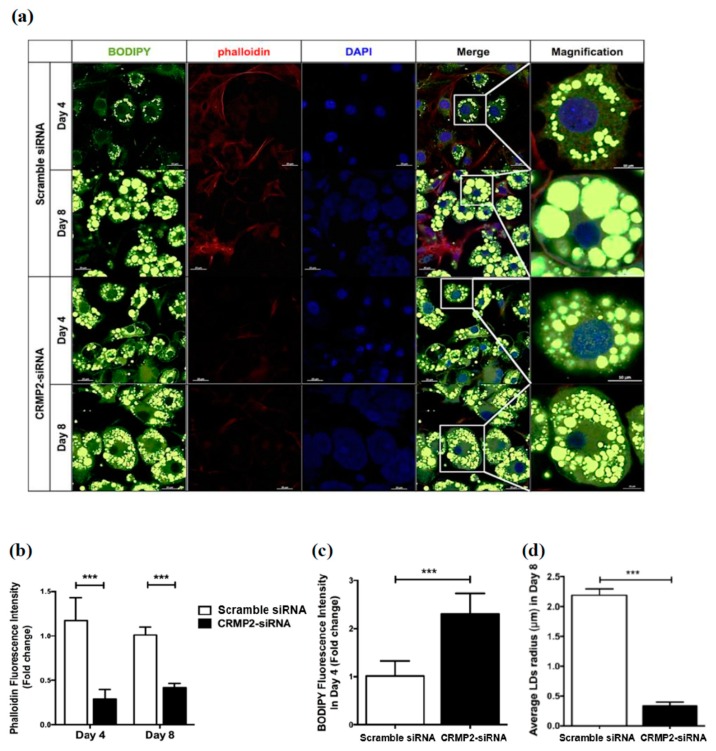
Effects of CRMP2 knockdown on cytoskeleton and lipid droplets. (**a**) Cells were transfected with CRMP2-specific siRNA (CRMP2-siRNA cells) or control siRNA (non-siRNA) on day -2, then subjected to differentiation. Lipid droplets (LDs) and phalloidin-positive actin filaments were detected on day 4 or day 8. (**b**–**d**) Quantitation of the signals in (**a**). The scale bars represented 20 μm in regular images and 10 μm in Magnification, respectively. Data were presented as means ± SEM of about 50 cells in each group, and analyzed by one-tailed unpaired Student *t*-test. *** *p* < 0.005.

The CRMP2-meidated cytoskeleton alterations coinciding with GLUT4 translocation implied the fusion between lipid droplets (LDs) during adipogenesis may also be affected. Therefore, LDs morphology in the middle (day 4) and late (day 8) stage of differentiation was microscopically investigated (Figure 7a). Phalloidin-staining F-actin intensity was significantly reduced in CRMP2-siRNA cells on day 4 and 8, indicating actin polymerization in the cortical area was affected (Figure 7b). CRMP2-siRNA cells contained significantly higher lipid contents on day 4 than their counterparts (Figure 7c), probably due to the elevated adipogenic transcription factors and lipid synthesis. Notably, while the LDs sizes in the CRMP2-siRNA cells were significantly larger on day 4, the mature adipocytes contained large amounts of smaller LDs despite they had higher lipid contents than the Control cells (Figure 7d). These findings suggest that although silencing CRMP2 leads to increased total lipid contents through upregulating the adipogenic transcription factors and lipid-synthesizing enzymes, the machinery responsible for GLUT4 translocation and LDs fusion is impaired by disrupting CRMP2-cytoskeleton interaction.

### 2.5. CRMP2 Expression Pattern Is Associated with Obesity

**Figure 8:**
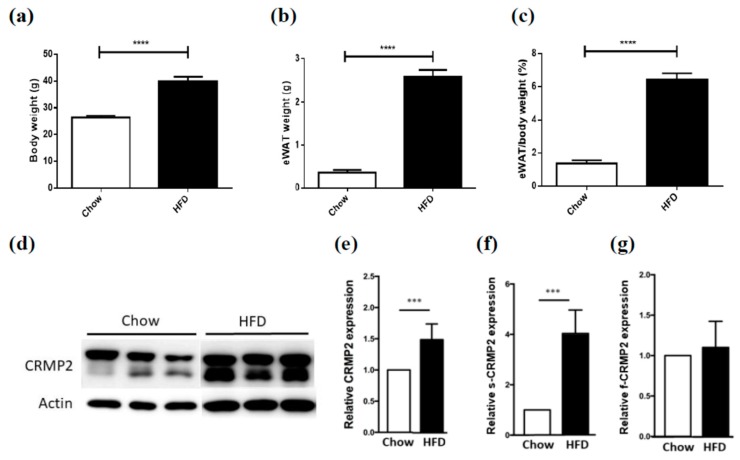
CRMP2 regulates adipocyte differentiation and lipid deposits. (**a**–**c**) Significant differences of body weights (BW), epididymal white adipose tissue (eWAT) weights and eWAT/ratio between Chow and HFD mice were identified. (**d**) Representative results of Western blot analysis and (**e**–**g**) quantitative results of CRMP2 expression in epididymal fat tissues obtained from standard Chow diet-feeding WT mice (*n* = 5) and HFD obese mice (*n* = 5), *** *p* < 0.001, **** *p* < 0.0001.

We next examined the in vivo CRMP2 expression profile to probe possible association between CRMP2 and obesity to verify the in vitro observations. The CRMP2 expression profile in epididymal adipose tissues harvested from diet-induced obese (HFD) and control (Chow) mice was examined. Significant differences regarding body weights (BW, 26.28 ± 0.93 vs. 39.94 ± 2.47 g, *p* < 0.0001; Figure 8a), epididymal white adipose tissue (eWAT) weights (0.36 ± 0.09 vs. 2.60 ± 0.23 g, *p* < 0.0001; Figure 8b), and eWAT/BW ratio (1.36 ± 0.30 vs. 6.43 ± 0.37%, *p* < 0.0001; Figure 8c) were observed between Chow and HFD mice. Total CRMP2 was significantly elevated in HFD mice (Figure 8d,e) primarily due to 4-fold increase of s-CRMP2 (Figure 8f) compared to the Chow counterparts, whereas, no significant alteration of f-CRMP2 levels between Chow and HFD mice was detected (Figure 8g). These data reveal that CRMP2 is involved in adipogenesis and associated with obesity.

## 3. Discussion 

Emerging epidemiological and molecular evidence indicates that neurodegenerative diseases and T2DM have common etiology and pathogenesis. Not only the levels of insulin and its receptor in AD patients are lower [26], but the hindered blood flow caused by persistent hyperglycemia in DM patients leads to cognitive impairment and dementia [27,28]. Therefore, T2DM is recognized as AD risk factor [29], and AD disease progression in T2DM patients is correlated with diabetic clinical manifestations [30]. Recently, hyperglycemia is reported to result in increased β amyloid and thus modulate neuronal activity [31], supporting that abnormalities in glucose metabolism contribute to AD pathogenesis and development [32]. In particular, AD is suggested to be designated as type 3 diabetes [26]. 

We recently added another molecular evidence supporting the convergence of pathogenesis leading to neurodegenerative and metabolic diseases by elucidating the involvement of PARL-PINK1-Parkin system in adipogenesis [17]. In the present study, we provide further evidence of the comorbidity between AD and diabetes by disclosing the involvement and regulation of adipocyte differentiation and lipid deposits by CRMP2. Being barely detected until the pre-adipocytes reached confluence, s-CRMP2 expression was gradually decreasing to an undetectable level in mature adipocytes (Figure 1). Whereas, the f-CRMP2 remained consistent during the entire differentiation period. The results indicate that CRMP2 isoforms and activity are coupled with the status of cell confluence, proliferation and differentiation.

The 58 kDa s-CRMP2 is generated by C-terminal processing [19]. In physiological condition, s-CRMP2 loses tubulin-binding activity and shows opposite function of f-CRMP2 by inhibiting neurite elongation. Similar to the decreasing expression pattern disclosed in the present study, s-CRMP2 is down-regulated to an undetectable level at late postnatal stages during brain development [19]. In pathological condition, s-CRMP2 is involved in controlling cell differentiation and proliferation, and therefore, correlated with poor prognosis in various cancers [7,10,14]. In support of the scenario in brain development, our finding concerning the coupling between s-CRMP2 expression and MCE stage indicates that the balance between f-CRMP2 and s-CRMP2 represents an important key in modulating cell proliferation at MCE stage in adipogenesis. The significantly increased adipose s-CRMP2 in HFD mice further support and indicate that s-CRMP2 is associated with obesity and may participate in diabetic pathogenesis and disease progression (Figure 8). Besides, CRMP2 activity is required in late stage of differentiation based on the undetected s-CRMP2 and pCRMP2 Thr514. 

Phosphorylation status is the other factor determining CRMP2 activity. The CRMP2-meidated neuronal morphological changes and growth polarity are closely associated with cytoskeleton remodeling [33,34,35]. CRMP2 co-localizes with F-actin in the growth cones of different types of neurons [9,36,37], and regulates axonal growth by promoting microtubule assembly through its interaction with tubulin [7]. The pCRMP2 significantly lowers its affinity for tubulin, loses tubulin-binding capacity and abolishes the ability to promote and sustain microtubule assembly, leading to tubulin depolymerization and microtubule destabilization. The downregulation of pCRMP2 Thr514 right after induction further supports our inference that CRMP2 activity is required for adipogenesis. Nevertheless, CRMP2 activity needs to be finely tuned since its overexpression inhibits cell proliferation and lipid synthesis (as discussed below).

After completing the task at MCE stage, differentiating cells are destined to express critical enzymes for lipid synthesis and accumulation for acquiring the characteristics of mature adipocytes. In addition to suppressing cell proliferation in MCE phase, overexpressed CRMP2 led to significantly reduced lipid deposits in mature adipocytes (Figure 2) by inhibiting the adipogenic transcription factors and lipid-synthesizing enzymes (Figure 3). These inhibitory effects were reversed by introducing CRMP2-siRNA (Figure 5). Notably, the insulin-induced glucose uptake was dramatically reduced although GLUT4 and insulin signaling were intact (Figure 6). Intriguingly, CRMP2-siRNA mature adipocytes contained large amounts of smaller LDs but higher lipid contents (Figure 7). 

Cytoskeleton plays an important role in the sequential evens of GLUT4 storage vesicles (GSVs) trafficking. Under insulin stimulus, GSVs travel along the microtubules from perinuclear area to cell cortex [38], followed by tethering, docking then fusing with the plasma membrane [39]. Lopez et al. [40] identified that the cortical actin polymerization and remodeling is a necessary and rate-limiting step for the fusion of GSVs with plasma membrane. This final GSVs fusion step is impaired when actin remodeling is disrupted, resulting in less GLUT4 molecules on plasma membrane while insulin-induced GSVs accumulation near the cortical area is intact. The authors not only demonstrate the roles of cytoskeleton and associated proteins in vesicle transportation, but characterize the final stage of GLUT4 translocation as an insulin-independent event [41]. Our study echoes the conclusion from Lopez et al. by revealing the involvement of CRMP2 in the insulin-independent GSVs fusion event via mediating cortical F-actin polymerization (Figure 6). Moreover, it suggests that the F-actin-involved GLUT4 translocation and thus glucose uptake efficiency is mediated by CRMP2 in an insulin-independent way.

Nobusue et al. also identified the cortical F-actin formation as an important event in adipogenesis [42]. The authors demonstrate that during the MCE phase of adipogenesis, F-actin stress fibers were first depolymerized to monomeric G-actin within 24 h after induction, then reorganized to form cortical F-actin structures within 48 h. This timeline of actin remodeling at MCE phase coincides with the s-CRMP2 expression (Figure 1), implying CRMP2 regulates adipocyte differentiation via mediating actin dynamics. We suggest that s-CRMP2 may perform as a dominant regulator over f-CRMP2 to direct cell proliferation and actin depolymerization for the cells to acquire the apparatus required for MCE phase mitosis. Then the s-CRMP2 is degraded when the cells exit from MCE phase, allowing f-CRMP2 to exert the functions modulating glucose uptake, lipid synthesis/accumulation, and morphological alterations in the late stage for the cells to achieve the differentiation mission. Accordingly, manipulating the balance between s-CRMP2 and f-CRMP2 and thus the cytoskeleton dynamics is very likely to improve the efficiency of glucose uptake and thus enhance insulin sensitivity. 

Except for the dramatic morphological changes for the fibroblastic pre-adipocytes transforming to the characteristic round-shape cells, mature adipocytes are laden with LDs as the energy reservoir. The de novo LD biogenesis is believed to be generated by budding of the progressively accumulated neutral lipids wrapped by ER membrane [43,44] despite several hypotheses regarding LD birth are proposed. Similar to GSVs trafficking, LDs move rapidly along microtubules and fuse each other to form larger droplets during maturation [45], particularly in the process of becoming the unilocular adipocytes. The disrupted cytoskeleton stability in CRMP2-siRNA cells contained higher lipid contents but large amounts of smaller LDs indicates that CRMP2-mediated cytoskeleton remodeling is involved in LD fusion. Furthermore, as the cortical F-actin is formed again in fully differentiated adipocytes and required for GLUT4 translocation, facilitating the formation of cortical F-actin is suggested to increase the microenvironmental mechanical strength, and therefore inhibit adipocyte hypertrophy in diet-induced obesity [46]. Our study supports this speculation by revealing that both glucose uptake and lipid accumulation are significantly impaired once the balance of CRMP2-mediated cytoskeleton dynamics is disturbed. We infer that, similar to the pathological role of s-CRMP2 in tumorigenesis, the highly expressed s-CRMP2 in HFD mice may thus play certain roles during the process of being obesity and eventually the diabetic onset. 

The results regarding increased s-CRMP2 levels in adipose tissue from obese animal models seem to be contradictory to the in vitro findings concerning decreasing s-CRMP2 pattern during differentiation and adipogenic-inhibitory effects of CRMP2. We suggest that the differential status of adipocytes in the cell and animal models should be able to explain these observations. While expression profiles of CRMP2 were temporally monitored to address the involvement of CRMP2 in the entire adipogenic process, the in vivo data represented the cross-sectional events of adipose CRMP2 levels in DIO mice. Accordingly, the in vitro findings demonstrate the participation and alterations of CRMP2 during the progression of adipocyte differentiation, while the animal data reveal the outcome in the hypertrophic adipose tissues after being obese. Notably, in support of previous report [16], the currently revealed association between CRMP2 and obesity provides another strong molecular evidence deciphering that CRMP proteins regulate energy metabolism in adipocytes.

Nevertheless, there are limitations in the present study. Firstly, the possibility of CRMP2 overexpression-induced apoptosis in MCE phase cannot be completely ruled out since the apoptotic cells in CRMP2-cells was not quantified. However, we supposed that the induced apoptosis, if any, should not contribute much and distort the conclusion. Besides, our current data are insufficient to provide the molecular mechanism regulating CRMP2 in adipogenesis. Once the pre-adipocytes enter differentiation, the actin stress fibers are rapidly depolymerized through downregulating RhoA-ROCK signaling, the upstream CRMP2-modulating molecule [47,48]. Accordingly, in addition to GSK-3β, RhoA-ROCK signaling pathway is very likely to play a role in regulating CRMP2 during adipogenesis. However, this speculation needs further study.

**Figure 9:**
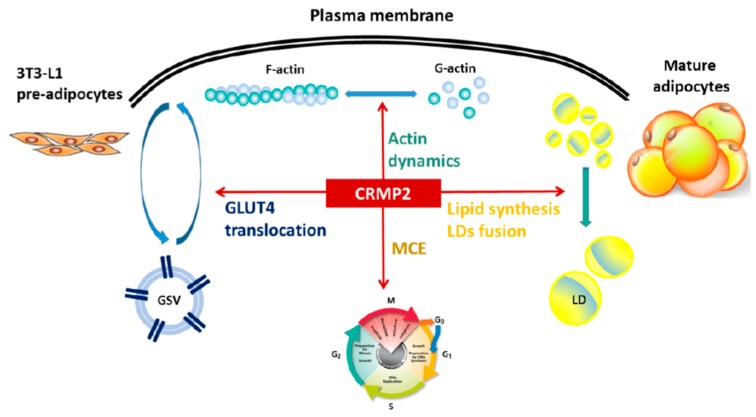
CRMP2 regulates adipocyte differentiation and lipid deposits. CRMP2 exerts multiple functions in mediating adipogenesis and determining lipid deposits in the mature adipocytes via [1] modulating cell proliferation at MCE phase; [2] regulating the expression of critical adipogenic transcription factors; [3] mediating the expression of adipogenic markers and lipid-synthesizing enzymes for lipid accumulation, and thus the lipid contents; and [4] modulating cytoskeleton polymerization required for GLUT4 translocation and LDs fusion. Accordingly, CRMP2 expression and function are correlated with the morphological changes and energy metabolism of adipocytes during adipogenesis, which determines the lipid deposits and therefore the mass of adipose reservoir. GSVs: GLUT4 storage vesicles; LD, lipid droplet.

Taking our data together, a model regarding multiple functions of CRMP2 in mediating adipocyte differentiation and lipid deposits is depicted (Figure 9). The s-CRMP2 expression is coupled with MCE phase to direct cell proliferation and cytoskeleton dynamics for the cells to acquire the mitotic apparatus. At the late adipogenic process, CRMP2 mediates glucose metabolism and lipid deposits via regulating (1) the expression of critical adipogenic transcription factors driving adipogenesis; (2) the expression of adipogenic markers and lipid-synthesizing enzymes for lipid accumulation, and thus the lipid contents; and (3) cytoskeleton remodeling required for morphological alterations, GLUT4 translocation and LDs fusion to achieve the goal of becoming mature adipocytes. In brief, CRMP2 expression and function are correlated with the morphological changes and energy metabolism in adipogenesis, and the lipid deposits. 

Given GSK-3β is the convergence between insulin signaling and cytoskeleton dynamics, it is logical to suggest that CRMP2 is a candidate gene for T2DM susceptibility as CRMP2-mediated cytoskeleton remodeling determines the mass of adipose reservoir which is associated with obesity and metabolic abnormalities. We suggest that the consequences of disrupting CRMP2 expression include glucose intolerance and obesity due to the inefficient glucose uptake but excess lipid synthesis and accumulation, which eventually would lead to the development of insulin resistance and diabetic onset. Furthermore, as persistent hyperglycemia causes increased contractility of vascular smooth muscle [49] and podocyte damage [50] via mediating actin polymerization, we speculate that CRMP2 dysregulation may also take part in diabetic disease progression and complications. At the best of our knowledge, this is the first report characterizing the functions of CRMP2 in adipogenesis for determining lipid deposits and possible implication in metabolic disorders. Moreover, our study provides further molecular evidence to support and explain the link of pathogenesis between neurodegenerative diseases and metabolic abnormalities. 

## 4. Materials and Methods

### 4.1. Reagents

Antibodies against CRMP2, phosphorylated CRMP2 (pCRMP2) Thr-514, CCAAT-enhancer-binding protein-α (C/EBPα), peroxisome proliferator-activated receptor gamma (PPARγ), adipocyte fatty acid binding protein-4 (FABP4), GSK-3β and phosphorylated GSK-3β (pGSK-3β) Ser-9 were purchased from Cell Signaling Technology (Danvers, MA, USA); glucose transporter 4 (GLUT4) from Novus Biologicals (Centennial, CO, USA); acetyl-CoA carboxylase 1 (ACC1), phosphorylated ACC1 (pACC1), diglyceride acyltransferase 2 (DGAT2), and GAPDH from GeneTex, Inc. (Irvine, CA, USA); fatty acid synthase (FAS) from BD Biosciences (San Jose, CA, USA); myc tag from Thermo Fisher Scientific (Waltham, MA, USA); and β-actin from Sigma (St. Louis, MO, USA). ECL reagent was purchased from Calbiochem (Merck Millipore, Billerica, MA, USA); Trizol Reagent from Life Technology (Carlsbad, CA, USA); 3-isobutyl-methylxanthine (IBMX), dexamethasone (Dex), and insulin from Sigma; non-specific scramble small interfering RNA (sramble siRNA, 5′-TTCTCCGAACGTGTCATGT-3′) and CRMP2-specific siRNA (Dypsl2-1: 5′-GGGAATGACATCCGCTGAT-3′; Dypsl2-2: 5′-CGGCTGAAGTCATCGCTCA-3′ and Dypsl2-3: 5′-GTTGAGAAGAGGCGGGTT-3′) purchased from BioTools, Inc. (Jupiter, FL, USA).; and DharmaFECT 1 reagent form Thermo Scientific DharmaFECT; Alexa Fluor 568 phalloidin and BODIPY^®^ 493/503 from Thermo Fisher Scientific.

### 4.2. 3T3-L1 Cell Culture, Adipogenesis, and Oil Red O Staining

3T3-L1 pre-adipocytes were maintained in DMEM containing 10% calf serum (Hyclone Laboratories, South Logan, UT, USA). Two day postconfluent 3T3-L1 cells (designated day 0) were induced to differentiate by addition of a standard cocktail (MDI) composed of 0.5 mM IBMX, 1 μM Dex, and 10 μg/mL insulin in 10% FBS for 2 days. The cells were then cultured in DMEM supplemented with 10% FBS and 5 μg/mL insulin. The medium was replaced by fresh medium every two days. Oil-Red O staining was performed as previously described [17,51,52]. For quantification, the dye was eluted by adding 100% isopropanol and the extracts were determined by measuring the absorbance at 490 nm.

### 4.3. CRMP2 Overexpression and Knockdown

CRMP2 mRNA was extracted from mature 3T3-L1 adipocytes and reverse-transcribed into cDNA. The CRMP2 gene fragments were amplified by myc taq-containing primers 5′-ATGTCTTATCAGGGGAAGAAAAATATTCCA-3′ and 5′-TTACAGATCTTCTTCAGAAATAAGTTTTTGTTCGCCCAGGCTGGTGATGT-3′) and subcloned into pTARGET^TM^ vector (Promega, Madison, WI, USA)) to generate CRMP2-carrying pTARGET^TM^ vector (pTARGET^TM^-CRMP2-Myc). For CRMP2 overexpression, cells were transfected with 1 μg of pTARGET^TM^-CRMP2-Myc using PolyJet^TM^ reagent (SignaGen Laboratories, Rockville, MD, USA) on day 2 and day 4, respectively, to analyze the effects of CRMP2 overexpression on the entire adipogenesis and lipid accumulation in the late adipogenic phase. For siRNA transfection, the pre-adipocytes were transfected with either 100 nM scramble siRNA or CRMP2 siRNA cocktail by DharmaFECT 1 reagent on day -2 according to the manufacturer’s instruction. The cells were incubated in medium containing the siRNA-DharmaconFECT1 complex for 16 h, and induced into differentiation after being incubated for another 24 h with fresh DMEM supplemented with 10% calf serum. 

### 4.4. Western Blot Analysis

Cell lysates were prepared in RIPA buffer containing protease inhibitors as described previously [17,51,52], subjected to SDS-PAGE, transferred to PVDF membrane and blotted with specific primary antibodies. For detection, membranes were incubated with secondary antibodies (Merck Millipore, Billerica, MA, USA) for 1 h, and results were visualized with ECL regent and exposed to X-films. The blot was quantified by Labscan software. 

### 4.5. Quantitative Real-Time PCR (qPCR) Analysis

Total RNA was isolated using Trizol Reagent. Briefly, complementary DNA was synthesized from 5 μg RNA using SuperScript III Reverse Transcriptase Kit (Invitrogen, Carlsbad, CA, USA). Target sequence-specific PCR primer sets included CRMP2: 5′-ATTCCAGCTGACGGATTCCCAGAT-3′ and 5′-TGATGTCACCATTCTCTGCGTGGA-3′; GAPDH: 5′-ACCACAGTCCATGCCATCAC-3′ and 5′-TCCACCACCCTGTTGCTGTA-3′. The PCR conditions consisted of pre-denaturation at 95 °C for 5 min followed by 35 cycles of amplification at 95°C for 15 s, 58 °C for 30 s, and 72 °C for 30 s, and a final 10-min extension step at 72 °C. 

### 4.6. Confocal Microscopy

Cells were fixed with 3.7% formaldehyde for 15 min and then permeabilized with 0.5% triton X-100 for 15 min at room temperature. The cells were then blocked with 5% BSA for 30 min at room temperature, followed by incubation with specific primary antibody overnight at 4 °C respectively. Goat anti-rabbit IgG conjugated DyLight^TM^594 (Jackson Immunoresearch Laboratories, West Grove, PA, USA) was added together with BODIPY and incubated for 1 h at room temperature. Cells were then mounted with GEL/MOUNT containing DAPI (Molecular Probes). The images were taken by ZEISS LSM 700 confocal fluorescence microscope system using 63X objective len.

### 4.7. Quantification of Glucose Uptake

Glucose uptake assay was performed as described [53,54] after cells were incubated with glucose-free DMEM for 8 h. Cells were fed with 250 µmol/L 2-[N-(7-nitrobenz-2-oxa-1,3-diazol-4-yl)amino]-2-deoxy-D-glucose (2-NBDG; Cayman Chemical, Ann Arbor, MI, USA) in the presence (INS) or absence (Mock) of insulin pre-treatment (100 nM for 30 min). Cellular 2-NBDG uptake was terminated after 10 min by adding ice-cold DMEM containing 10 mM glucose. Cells were washed with ice-cold PBS and lysed, and intracellular fluorescence intensity was measured (485⁄540 nm, Infinite 200).

### 4.8. Measurement of GLUT4 Translocation

Cells were treated with 100 nM insulin for 30 min after 2 h serum starvation, fixed with 4% paraformaldehyde and incubated with anti-GLUT4 for 1 hr at 4 °C, followed by adding secondary antibodies (Merck Millipore, Billerica, MA, USA) after washing. Then o-phenylenediamine dihydrochloride-containing phosphate-citrate buffer (0.05 M) was added, and absorbance at 450 nm was measured and quantitated (Infinite 200). The difference of GLUT4 fluorescent intensity between the control and insulin-treated cells indicated the increased insulin-induced GLUT4 on the cell membrane, which represented the amount of GLUT4 translocation in the presence of insulin stimulation.

### 4.9. Animal Experiments

Animal experiments were conducted as described [17,51,52,53,54,55]. In brief, 4-week-old male C57BL/6 mice were fed with high fat diet (HFD) or standard chow diet (Chow) for 8 weeks. Protein extracts from the harvested primary epididymal fat tissues were obtained by homogenizing the tissues using T-PER tissue protein extraction reagent (Pierce, Rockford, IL, USA) supplied with phosphatase and protease inhibitors (Roche, Indianapolis, IN, USA), then subjected to Western analysis. Animal protocols were reviewed and approved by the Institutional Animal Care and Use Committee, Hungkunag University.

### 4.10. Statistical Analysis

Each experiment was carried out at least three times. Results were presented as mean ± S.E.M. and the significant difference between groups was analyzed by two-tailed unpaired Student *t*-test, one-way or two-way ANOVA. Statistical difference was defined as *p* < 0.05 for all tests.

## Abbreviations

ACC	acetyl-CoA carboxylase
AD	Alzheimer’s disease
ALLN	calpain inhibitor Ac-Leu-Leu-Nle-CHO
Cdk5	cyclin-dependent kinase 5
C/EBPα	CCAAT-enhancer-binding protein-α
CRMP2	collapsin response mediator protein 2
Dex	dexamethasone
DGAT	diglyceride acyltransferase
FABP4	adipocyte fatty acid binding protein-4
FAS	fatty acid synthase
GLUT4	glucose transporter 4
GS	glycogen synthase
GSK-3β	glycogen synthase kinase-3β
GSVs	GLUT4 storage vesicles
IBMX	3-isobutyl-methylxanthine
PP	lambda protein phosphatase
LDs	lipid droplets
MCE	mitotic clonal expansion
PD	Parkinson’s disease
PI3K	phospho-inositide 3-kinase
PPARγ	peroxisome proliferator-activated receptor gamma
siRNA	small interfering RNA
T2DM	type 2 diabetes mellitus
